# Taccalonolides: Structure, semi-synthesis, and biological activity

**DOI:** 10.3389/fphar.2022.968061

**Published:** 2022-08-11

**Authors:** Yan Li, Yu-Feng Du, Feng Gao, Jin-Bu Xu, Ling-Li Zheng, Gang Liu, Yu Lei

**Affiliations:** ^1^ Department of Pharmacy, The First Afflicted Hospital of Chengdu Medical College, Chengdu, China; ^2^ School of Life Science and Engineering, Southwest Jiaotong University, Chengdu, China

**Keywords:** taccalonolides, microtubule-stabilizer, structural classification, antitumor, pharmacological mechanism

## Abstract

Microtubules are the fundamental part of the cell cytoskeleton intimately involving in cell proliferation and are superb targets in clinical cancer therapy today. Microtubule stabilizers have become one of the effectively main agents in the last decades for the treatment of diverse cancers. Taccalonolides, the highly oxygenated pentacyclic steroids isolated from the genus of *Tacca*, are considered a class of novel microtubule-stabilizing agents. Taccalonolides not only possess a similar microtubule-stabilizing activity as the famous drug paclitaxel but also reverse the multi-drug resistance of paclitaxel and epothilone in cellular and animal models. Taccalonolides have captured numerous attention in the field of medicinal chemistry due to their variety of structures, unique mechanism of action, and low toxicity. This review focuses on the structural diversity, semi-synthesis, modification, and pharmacological activities of taccalonolides, providing bright thoughts for the discovery of microtubule-stabilizing drugs.

## Introduction

**FIGURE 1:**
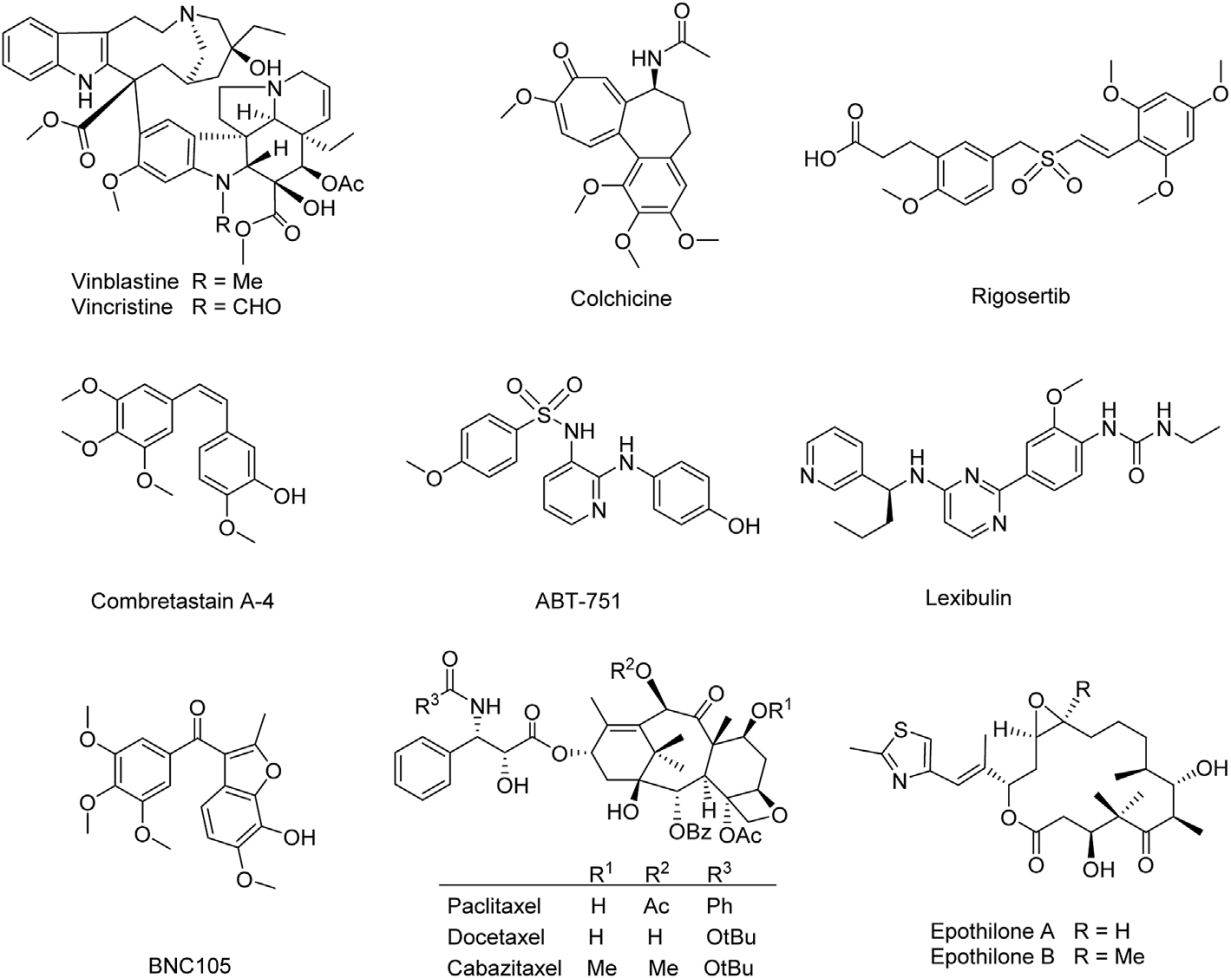
Microtubule-targeting agents for the treatment of cancer.

Natural products and their derivatives have been severed as an unsurpassed source to find microtubule-targeting drugs. Numerous natural-related microtubule-targeting agents currently in discovery or approved in preclinical or clinical are classified into two groups: microtubule-destabilizing agents (e.g., vinblastine, vincristine, colchicine, rigosertib, combretastatin A-4, ABT-751, lexibulin, and BNC105) (Taylor, 1965; Bryan, 1971; Griggs et al., 2001; Yee et al., 2005; Kremmidiotis et al., 2010; Lickliter et al., 2010; Jost et al., 2017), and microtubule-stabilizing agents (e.g., paclitaxel, docetaxel, cabazitaxel, and epothilones A and B) (Figure 1) (Bhalla et al., 1993; Jordan, 2002; Fojo and Menefee, 2007; Ojima et al., 2014), depending on their different mechanisms. Though these anti-mitotic agents have been utilized for the clinical treatment of different cancerous patients in the last decades, high toxicity, poor solubility, low oral bioavailability, and multidrug resistance render these agents less optimum for the clinical treatment of cancer (Gottesman et al., 1996; Litman et al., 2001; Zhao et al., 2016). Therefore, it remains essential to develop new microtubule-targeting agents with fewer side effects and improved activity against various classes of tumors.

The pentacyclic steroids taccalonolides are isolated from plants *Tacca plantaginea* (Hance) Drenth, *Tacca chantrieri* Andre, and *Tacca paxiana*. Taccalonolides are the first plant-derived microtubule-stabilizing agents except for paclitaxel. They are also the first natural steroids with microtubule-stabilizing activity (Liu et al., 2015). The previous reviews illustrated that over 120 constituents had been isolated from the genus *Tacca*, including 33 kinds of taccalonolides (Jiang et al., 2014; Li et al., 2014). Chen *et al.* and Risinger *et al.* summarized the biological activities of taccalonolides, as well as the challenges in formulation and supply (Risinger and Mooberry, 2010; Chen et al., 2021). Viewing the importance of the relationships between structural characteristics and biological activity, the present review is mainly focused on the classification of taccalonolides, as well as the semisynthetic taccalonolides and their synthetic routes. All the natural taccalonolides are classified into three categories according to structural characteristics and discussed in detail, along with the semisynthetic taccalonolides and their synthetic routes are summarized for the first time. The biological activities of taccalonolides in recent 6 years and the structure-activity relationships are also included.

## The chemical structures of taccalonolides

In the early 1960s, Professor Paul Scheuer investigated the “bitter principle” of the tubers of *T. leontopetaloides*, a starchy food source. A compound named taccalin was purified. As an intensely bitter, this light-yellow powder had a probable tetracyclic structure (Scheuer et al., 1963). The actual structure of taccalonolides was later found to be much larger, but this pioneering work laid the groundwork for the elucidation of their structures. Up to now, 41 natural taccalonolides have been isolated from the genus *Tacca* (Table 1).

**TABLE 1:** The name and source of natural taccalonolides.

Compd	Name	Source	References
1	Taccalonolide A	*T. plantaginea and T. paxiana*	Chen et al. (1987) and Muhlbauer et al. (2003)
2	Taccalonolide B	*T. plantaginea and T. paxiana*	Chen et al. (1987) and Muhlbauer et al. (2003)
3	Taccalonolide C	*T. plantaginea*	Chen et al. (1988a)
4	Taccalonolide D	*T. plantaginea*	Chen et al. (1988b)
5	Taccalonolide E	*T. plantaginea and T. paxiana*	Muhlbauer et al. (2003) and Shen et al. (1991)
6	Taccalonolide F	*T. plantaginea*	Shen et al. (1991)
7	Taccalonolide G	*T.plantaginea*	Chen et al. (1997)
8	Taccalonolide H	*T. plantaginea*	Chen et al. (1997)
9	Taccalonolide I	*T. plantaginea*	Chen et al. (1997)
10	Taccalonolide J	*T. plantaginea*	Chen et al. (1997)
11	Taccalonolide K	*T. plantaginea and T. paxiana*	Muhlbauer et al. (2003) and Chen et al. (1997)
12	Taccalonolide L	*T. plantaginea*	Shen et al. (1996)
13	Taccalonolide M	*T. plantaginea*	Shen et al. (1996)
14	Taccalonolide N	*T. paxiana*	Muhlbauer et al. (2003)
15	Taccalonolide O	*T. subflaellata*	Huang et al. (2002)
16	Taccalonolide P	*T. subflaellata*	Huang et al. (2002)
17	Taccalonolide Q	*T. subflaellata*	Huang et al. (2002)
18	Taccalonolide R	*T. paxiana, T. chantrieri, T. integrifolia*	Muhlbauer et al., 2003; Peng et al., 2011
19	Taccalonolide S	*T. paxiana*	Muhlbauer et al. (2003)
20	Taccalonolide T	*T. paxiana, T. chantrieri, T. integrifolia*	Muhlbauer et al., 2003; Peng et al., 2011
21	Taccalonolide U	*T. paxiana*	Muhlbauer et al. (2003)
22	Taccalonolide V	*T. paxiana*	Muhlbauer et al. (2003)
23	Taccalonolide W	*T. plantaginea*	Yang et al. (2008)
24	Taccalonolide X	*T. plantaginea*	Yang et al. (2008)
25	Taccalonolide Y	*T. plantaginea*	Yang et al. (2008)
26	Taccalonolide Z	*T. chantrieri, T. integrifolia*	Peng et al. (2011)
27	Taccalonolide AA	*T. chantrieri, T. integrifolia*	Peng et al. (2011)
28	Taccalonolide AB	*T. chantrieri, T. integrifolia*	Peng et al. (2011)
29	Taccalonolide AC	*T. plantaginea*	Li et al. (2011)
30	Taccalonolide AD	*T. plantaginea*	Li et al. (2011)
31	Taccalonolide AE	*T. plantaginea*	Li et al. (2011)
32	Taccalonolide AF	*T. plantaginea*	Li et al. (2011)
33	Taccalonolide H2	*T. plantaginea*	Li et al. (2011)
34	Taccalonolide AT	*T. chantrieri*	Ni et al. (2015)
35	Taccalonolide AU	*T. chantrieri*	Ni et al. (2015)
36	Taccalonolide AV	*T. chantrieri*	Ni et al. (2015)
37	Taccalonolide AW	*T. chantrieri*	Ni et al. (2015)
38	Taccalonolide AX	*T. chantrieri*	Ni et al. (2015)
39	Taccalonolide AY	*T. chantrieri*	Ni et al. (2015)
40	Taccalonolide AI	*T. chantrieri*	Peng et al. (2014)
41	Taccalonolide AG	*T. plantaginea*	Risinger et al. (2011)

**FIGURE 2:**
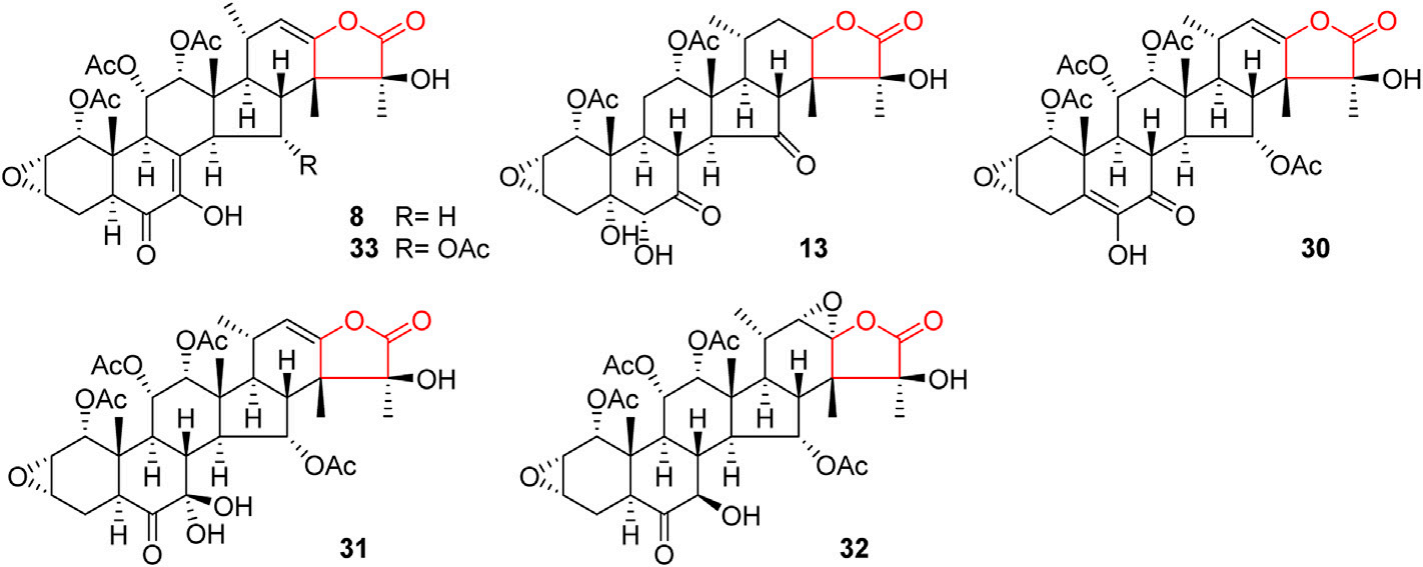
Structures of taccalonolides with lactone ring at C23–C26.

**FIGURE 3:**
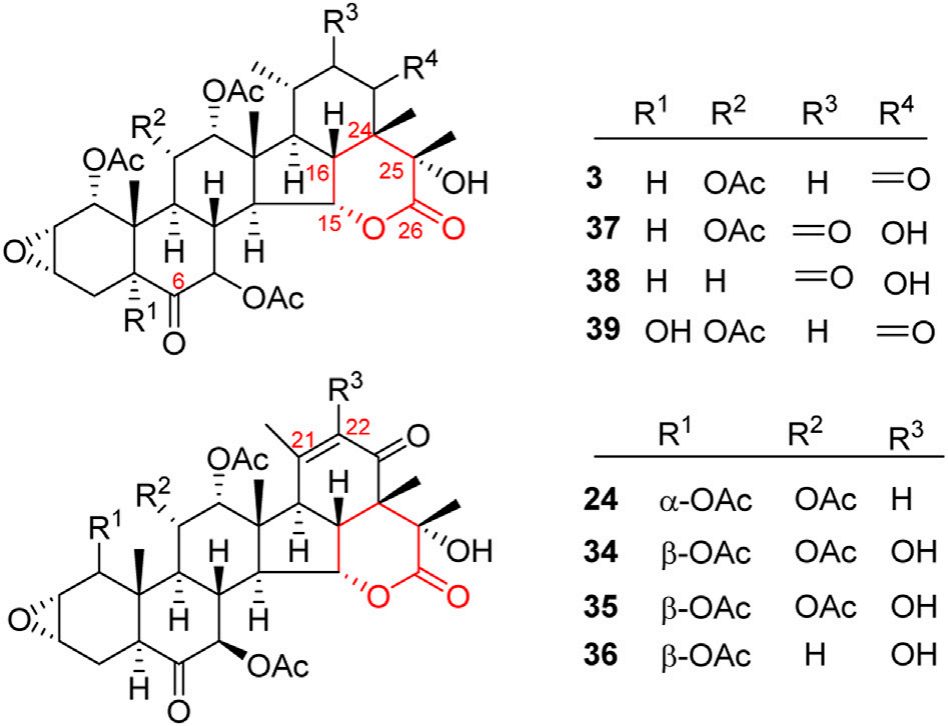
Structures of taccalonolides with lactone ring at C15–C26.

**FIGURE 4:**
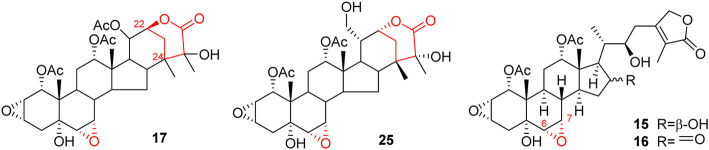
Structures of taccalonolides with lactone ring at C22–C24 and others.

Like general steroids, most of taccalonolides are based on a pentacyclic carbon framework. The unique structural feature of taccalonolides that they contain an epoxy function between C2 and C3. In the present review, taccalonolides are mainly divided into four categories according to the different positions of the lactone ring in the structures. The first category is represented by taccalonolide A (**1**, Figure 2) isolated from *T. plantaginea* and is the largest number of taccalonolides. The characteristic feature of this class is structural lactone rings linked to C23 and C26. The second category is represented by taccalonolide AT (**34**, Figure 3). Chemical structure of the second category is similar to the first class except for the six-membered lactone ring bearing the C15–C26 bond. The third classification is relatively small and represented by taccalonolide O (**17**, Figure 4). It has the hexacyclic feature and the same class of carbon skeleton with formers, characterized by a C22–C24 lactone ring. Besides, taccolanolides O (**15**) and P (**16**) (Figure 4) are classified into the last group. They have the characteristics of taccalonolide, though the partial carbon skeletons have changed.

### C23–C26 lactone ring taccalonolides

In 1987, taccalonolides A and B (**1** and **2**) were firstly found in the rhizome of *T. plantaginea* (Chen et al., 1987). Later, nine related compounds taccalonolides D–L (**4**–**12**) were reported from the same plant*.* They all contain a double bond between C22 and C23 (Chen et al., 1988a; Shen et al., 2010; Shen et al., 1996; Chen et al., 1997; Muhlbauer et al., 2003). Taccalonolide N obtained from *T. paxiana* possessed a similar structure. Taccalonolides H and H2 (**8** and **33**) feature an unusual double bond at C7–C8. Unlike other taccalonolides belonging to this class, taccalonolide M (**13**) and taccalonolide AF (**32**) do not possess any double bonds. Chemical investigation of the roots of *T. paxiana* resulted in the isolation of five new steroidal compounds, taccalonolides R–V (**18**–**22**) (Muhlbauer et al., 2003). From the whole plants of *T. plantaginea*, five new pentacyclic steroids, taccalonolides W (**23**) and AC–AF (**29**–**32**) were isolated (Yang et al., 2008; Li et al., 2011). Taccalonolide AF (**32**) is the only one in this category with a C22–C23 epoxy ring and is presumably derived from the epoxidation of taccalonolides A (**1**). Peng *et al.* obtained three new taccalonolides Z (**26**), AA (**27**), and AB (**28**) from *T. chantrieri* and *T. integrifolia* (Peng et al., 2011), while taccalonolides AI (**40**) and AG (**41**) were reported from the roots and rhizomes of *T. chantrieri* (Risinger et al., 2011; Peng et al., 2014). All compounds are substituted with a five-membered lactone ring connected in positions C23 and C26, which is the important feature of taccalonolides in this class (Figure 2).

### C15–C26 lactone ring taccalonolides

A six-membered lactone ring located in positions C15 and C26 features the characteristics of taccalonolides in this class. Taccalonolide C (**3**), the first example of this type, was isolated from the rhizome of *T. plantaginea*. Taccalonolide C (**3**) might derive from taccalonolide D (**4**): the C23–C24 lactone ring of **4** opens and then reforms a new lactone ring with C15 hydroxyl group (Chen et al., 1988b). Taccalonolides AW–AY (**37**–**39**) were isolated from the ethanolic extract of the whole plants of *T. chantrieri* (Ni et al., 2015). All these taccalonolides have a ketone group in position C6. Some compounds also contain a double bond in positions C21–C22, e.g., taccalonolides X (**24**) and AT–AV (**34**–**36**) (Figure 3) (Yang et al., 2008; Ni et al., 2015).

### C22–C24 lactone ring taccalonolides

The last category possesses a six-membered lactone ring connected in positions C22 and C24. The representative is taccalonolide Q (**17**) from the rhizomes of *T. subflaellata* (Huang et al., 2002). Taccalonolide Y (**25**) was firstly isolated from the whole plants of *T. plantaginea* (Figure 4) (Yang et al., 2008). In addition to the common epoxy function between C2 and C3, the third type of taccalonolides also have an extra epoxy in positions C6 and C7, along with a hydroxyl group at C5.

### Other taccalonolides

Two novel steroidal bitter principles, taccolanolides O (**15**) and P (**16**) are isolated from the tubers of *T. subflaellata* (Huang et al., 2002)*.* Differing from the above-mentioned taccolanolides, they do not contain a six-membered E ring in the structure. An epoxy group presents in positions C6 and C7.

## Semi-synthesis and modification of taccalonolides

**FIGURE 5:**
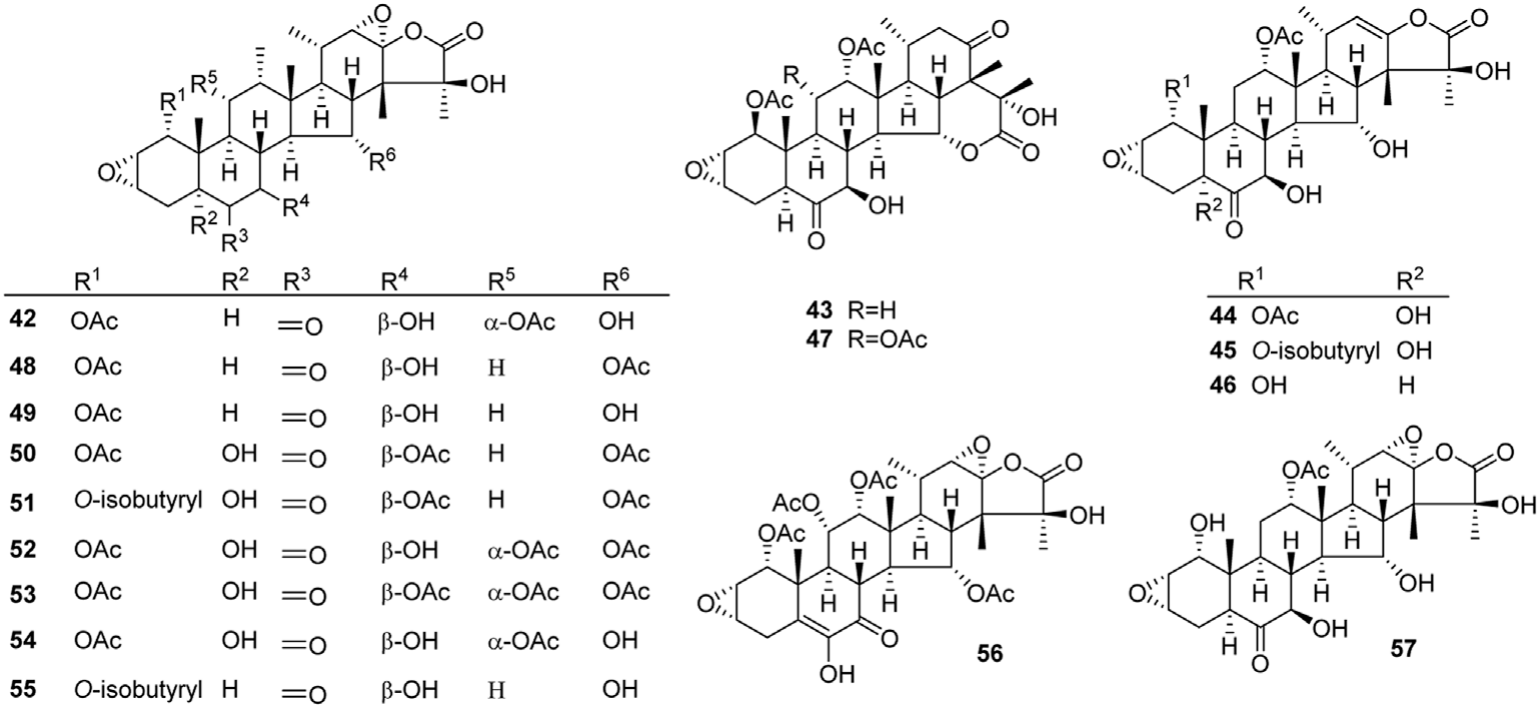
Structures of semi-synthetic taccalonolides.

**FIGURE 6:**
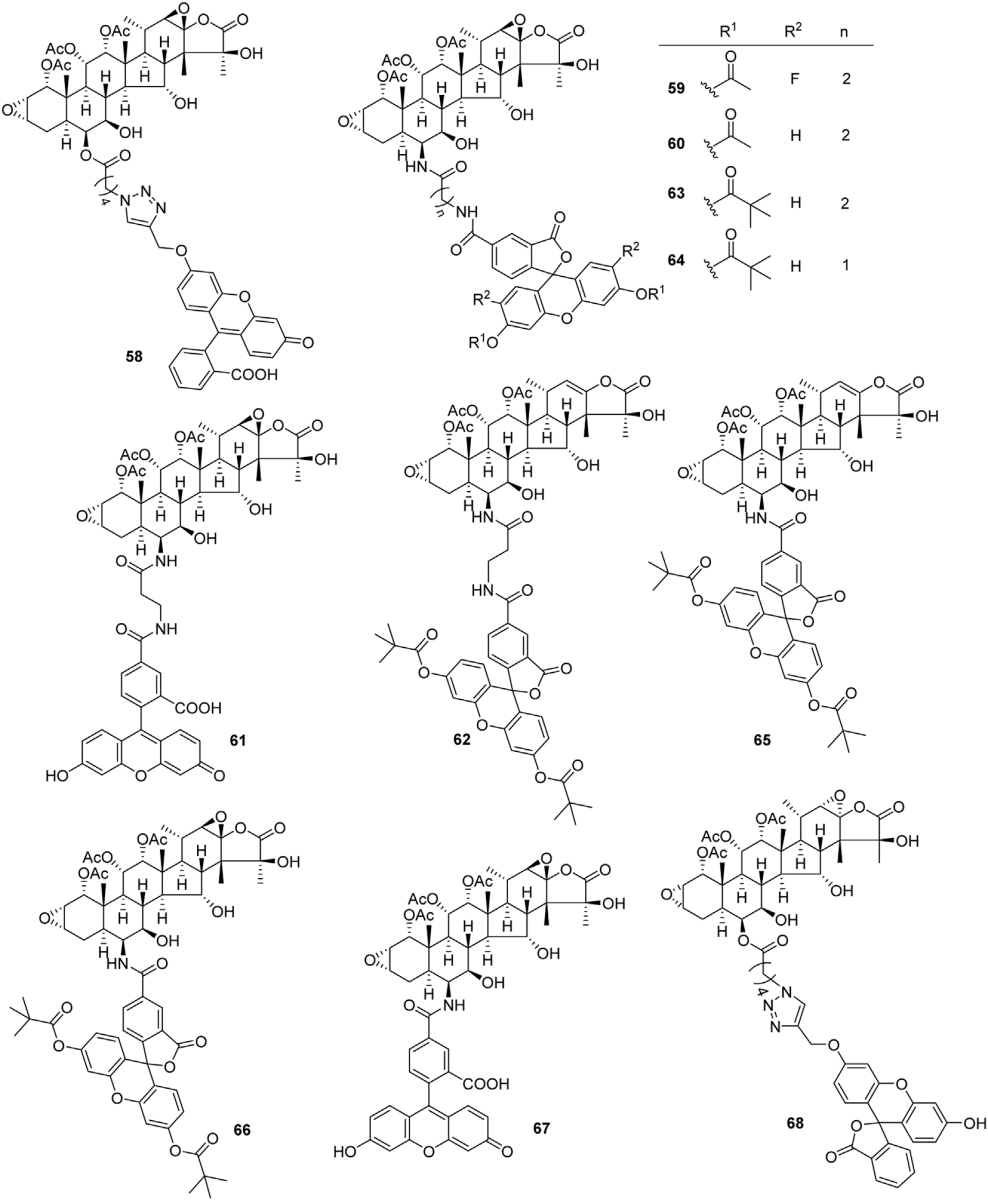
Structures of synthetic taccalonolides probes.

Taccalonolides are rare in nature, which is one obstacle to developing taccalonolides into drugs. Due to complex structures, multiple fragile functional groups, and high costs of synthesis, the total synthesis of taccalonolides has not been reported until now. Semi-synthesis may solve the problem of resource shortage. There are a few semi-synthetic taccalonolides. The reported structural modifications of taccalonolides include simple hydrolysis, epoxidation, and attaching a fluorescein group at C6. So far, twenty-seven taccalonolides and their derivatives were obtained by semi-synthesis (Figures 5, 6).

**SCHEME 1:**
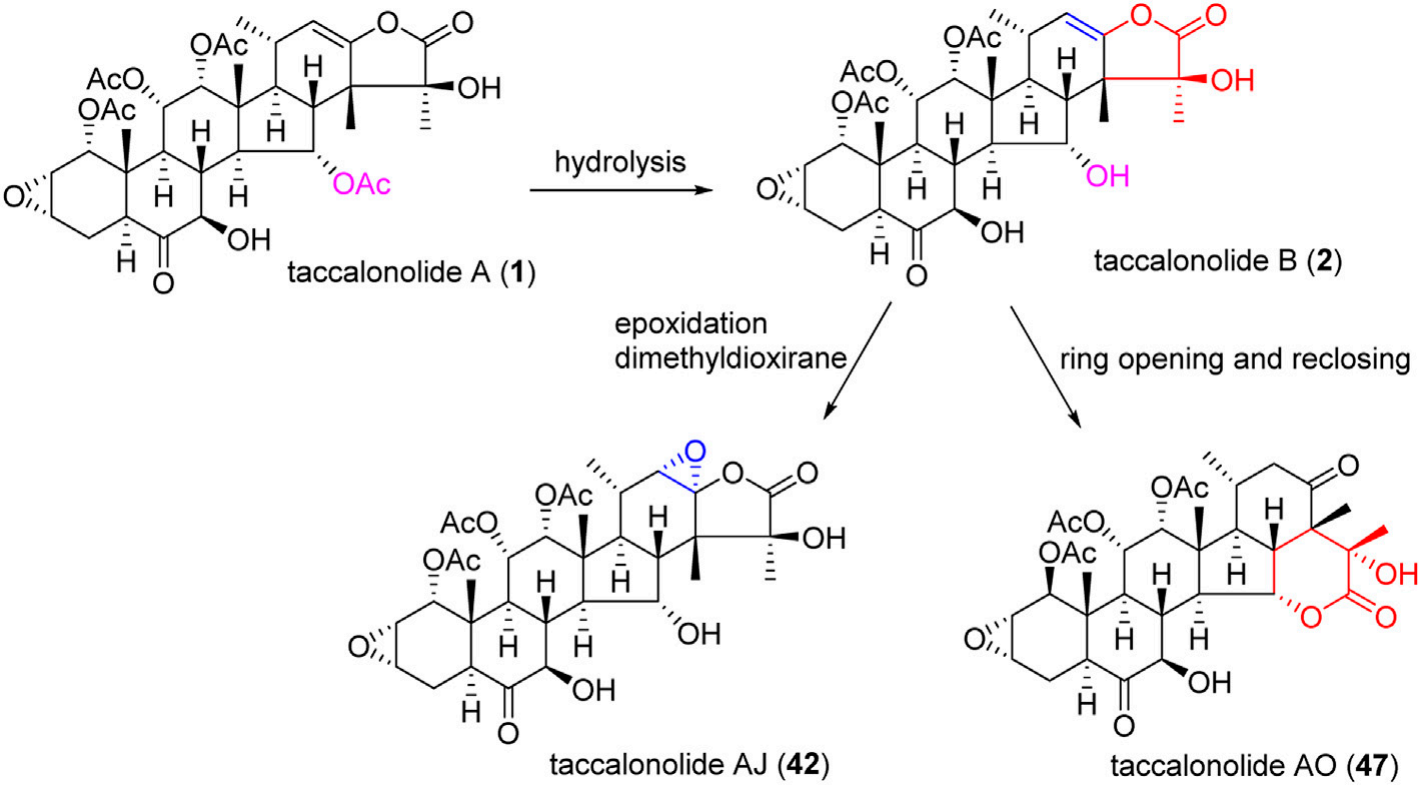
Synthetic route of taccalonolide AJ and AO.

**SCHEME 2:**
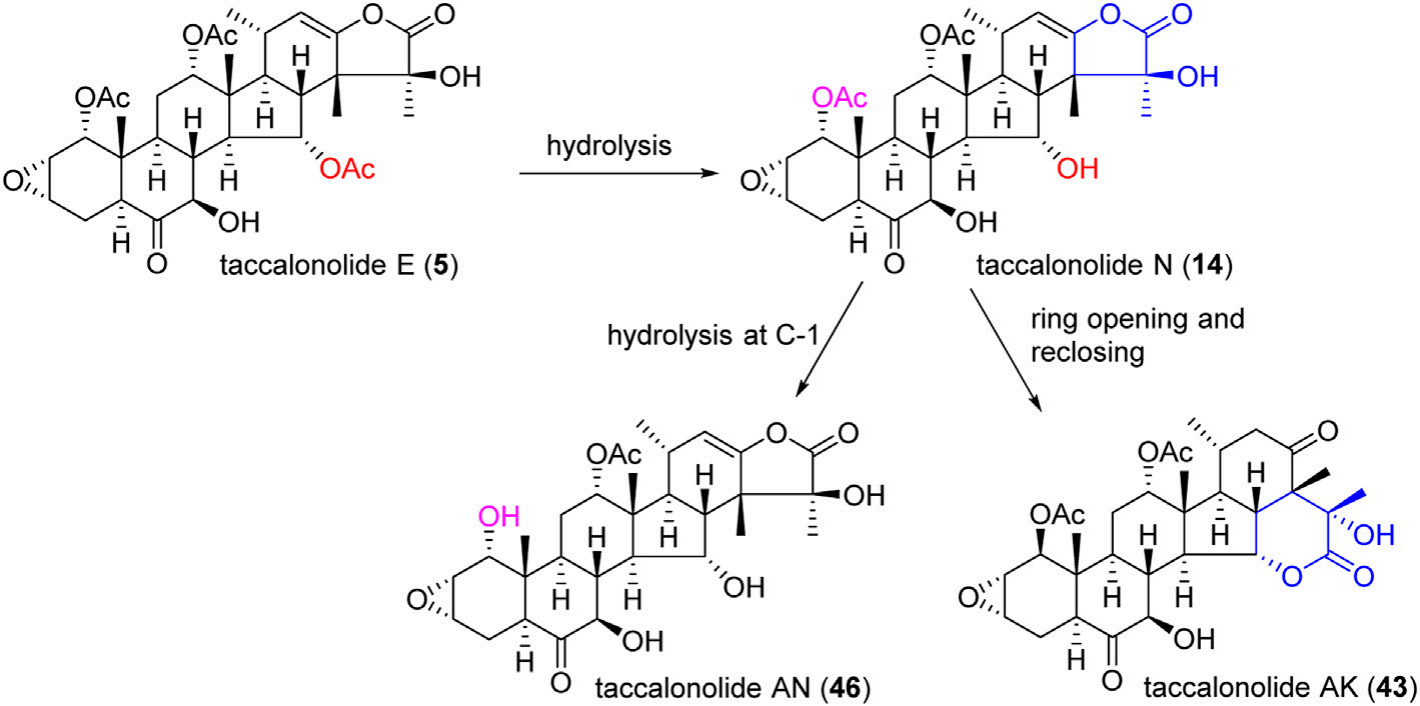
Synthetic route of taccalonolides AN and AK.

**SCHEME 3:**
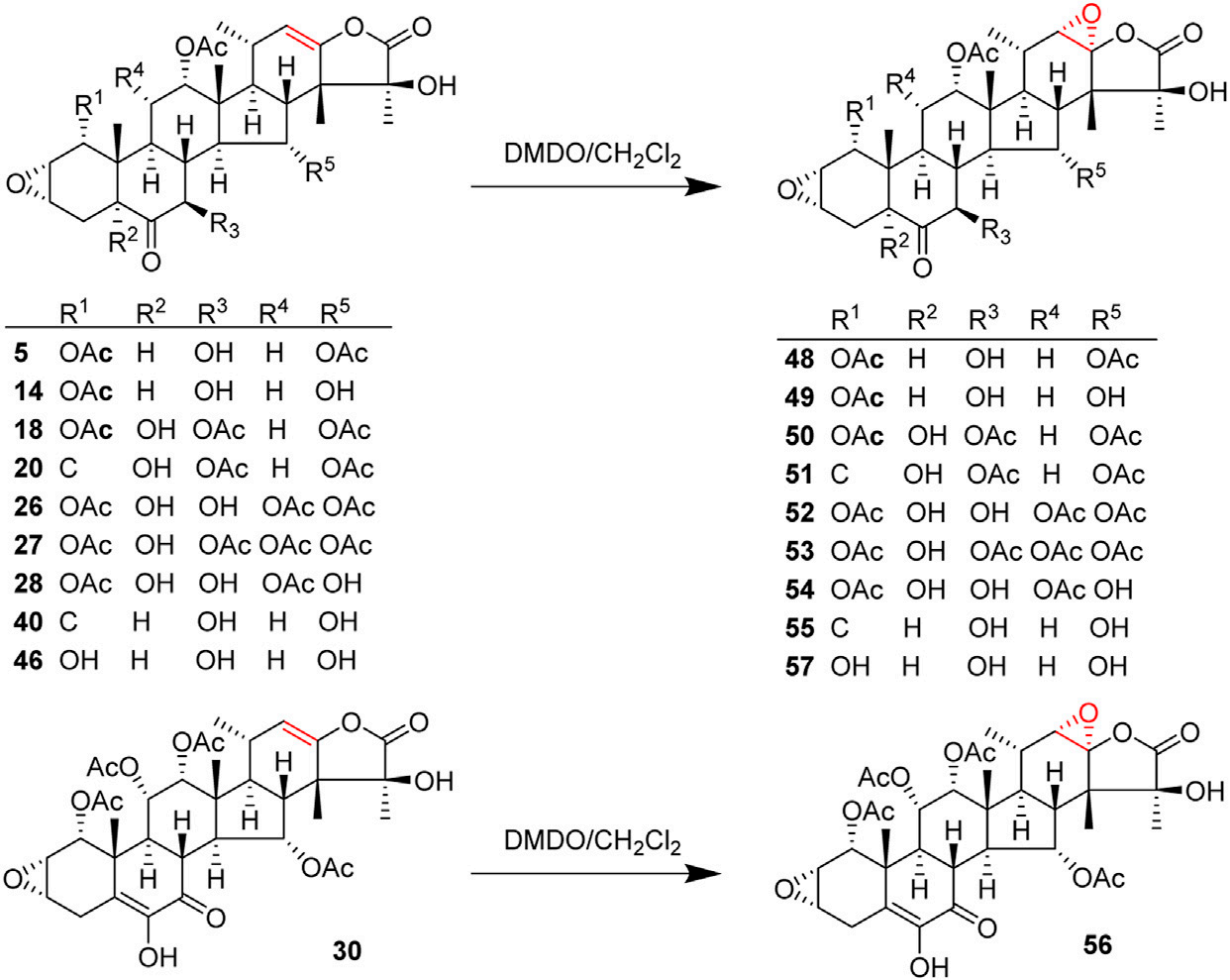
Synthetic route of epoxidation of taccalonolides.

An epoxidation reaction of compound **1** affords **32**, which leads to a drastic increase in antiproliferative potency (Adam et al., 2004). Li *et al.* performed the same epoxidation reaction on taccalonolide B to generate taccalonolide AJ (**42**) via two-step reactions, namely hydrolysis of taccalonolide A to obtain taccalonolide B, and epoxidation of taccalonolide B to form an epoxy group in positions C22 and C23, along with a new minor compound, designated taccalonolide AO (**47**), was obtained (Scheme 1) (Li et al., 2011). Similarly, taccalonolide N (**14**) could be obtained from the hydrolysis of taccalonolide E (**5**). Followed by hydrolysis at C1 or lactone ring-opening and re-closing, taccalonolides AN (**46**) and AK (**43**) were yielded, respectively (Scheme 2) (Li et al., 2013). DMDO (3,3-dimethyldioxirane) is an efficient and mild reagent, which can rapidly epoxidize taccalonolides under neutral and mild conditions. Epoxidation of C22–C23 double bond in 5, 14, 18, 20, 26–28, 30, 40, and 46 by DMDO could quantitatively yield 48–57, respectively (Scheme 3) (Peng et al., 2014).

**SCHEME 4:**
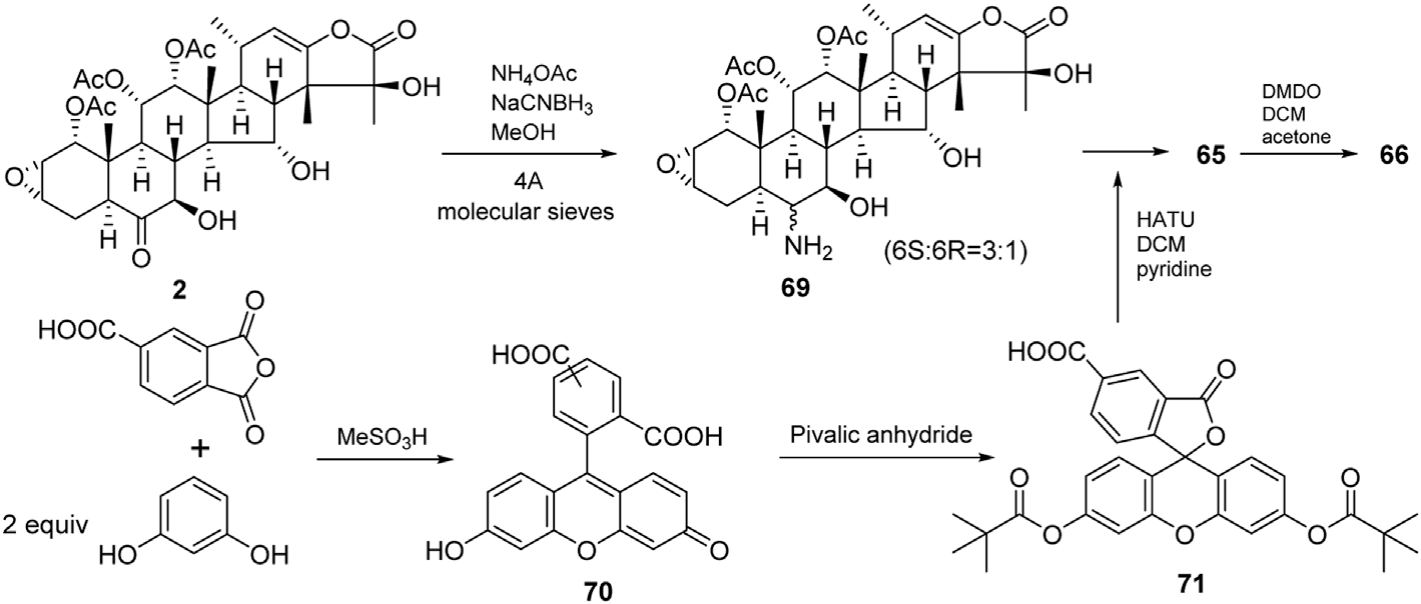
Synthetic routes of **65** and **66**.

**SCHEME 5:**
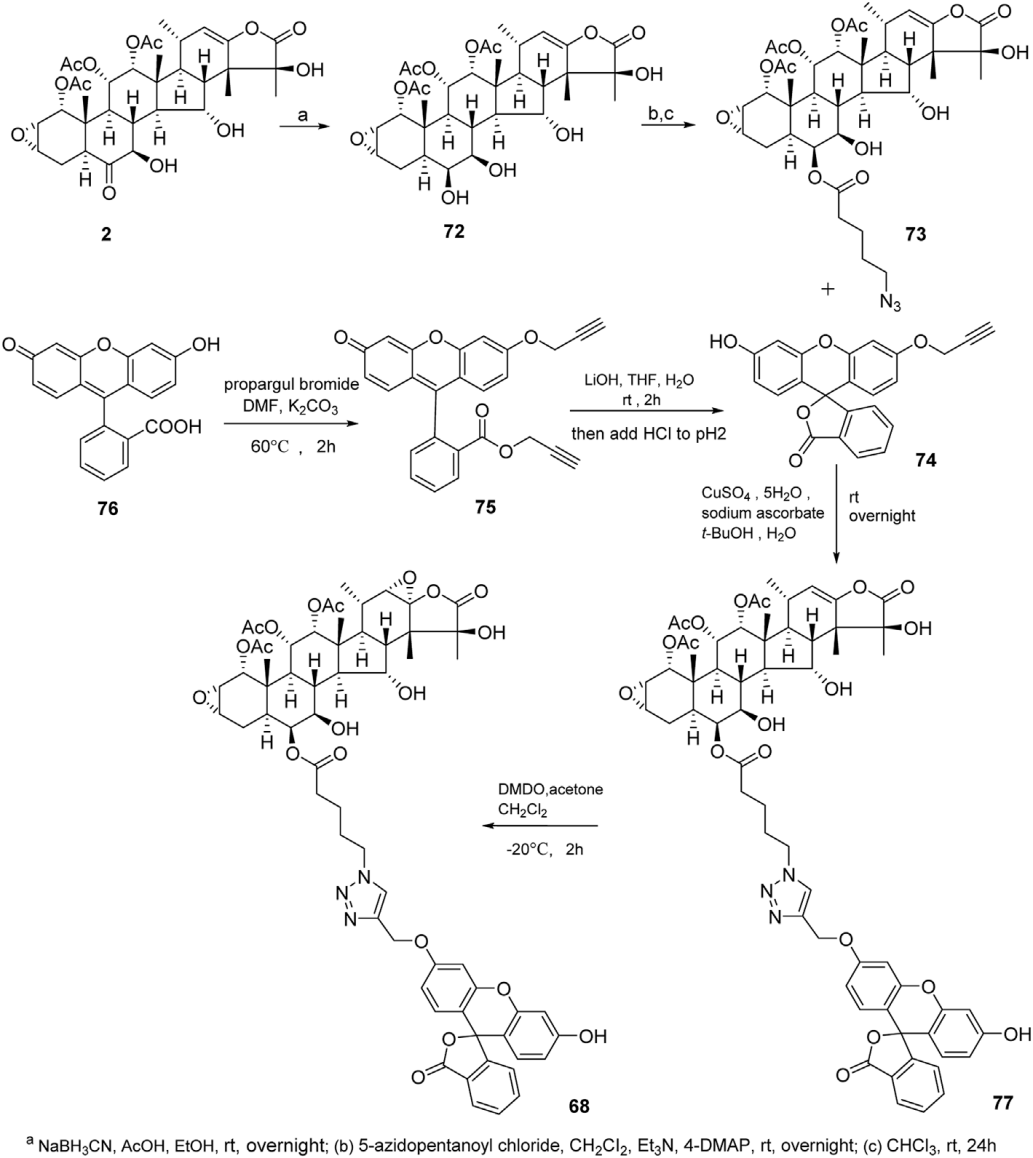
Synthetic route of **68**.

Taccalonolides cannot dissociate from tubulin and be detected after binding. The biochemical and pharmacokinetic analyses are limited by the covalent bonding. Inspired by cell-permeable taxane-based probes, Du *et al.* reported the fluorogenic taccalonolide probes that maintain the native biological properties of **42**, allowing for more detailed evaluations of the uptake, target binding, and distribution of these compounds *in vitro* and *in vivo* (Du et al., 2019; Du et al., 2020). When the fluorescein moiety links with the taccalonolide skeleton by an amide bond or ester bond in C6 position, their microtubule-binding and -stabilizing activities will not be compromised. Totally, eleven fluorogenic taccalonolide probes (58–68) were obtained (Figure 6). Among them, dipivaloyl-protected compound 66 is the most potent irreversible fluorogenic microtubule probe. The synthetic routes of 65 and 66 are shown in Scheme 4. Apart from the amide-linked fluorescein tag in C6 position, one more ester linker exists in their structures. The reduction of taccalonolide B (2) by NaBH_3_CN results in the stereospecific formation of *6S*–OH derivative, which is ideally suited for esterification. Du *et al.* also synthesized the fluorescein derivatives with an alkyne linker. The derivatives were covalently bonded via “click” chemistry to generate the intermediate 77. Epoxidation of C22–C23 double bond in 77 affords 68 (Scheme 5).

## Biological activity of taccalonolides

The structural diversity of taccalonolides leads to the diversity of biological activity, which mainly manifests in microtubule-stabilizing, cytotoxic activity, antitumor, drug-sensitive, and multidrug-resistant cell lines. According to the biological activities *in vitro* and *in vivo*, Risinger *et al.* clearly summarized the biological activities of taccalonolides before 2010 (Risinger and Mooberry, 2010). The current review concerns the biological activities of taccalonolides from 2010 (Table 2). Notably, the research on the mechanism of taccalonolides that made a breakthrough is focused.

**TABLE 2:** The biological activity of taccalonolides in this review.

Compounds	Model/Cell	Activity	References
1, 2, 5, 14, 18, 20, 26–28, 30–33, 42, 44–46, 51, 55	HeLa cells	microtubule-stabilizing activity	Peng et al., 2010
			Yang et al., 2008
			Li et al., 2011
			Peng et al., 2014
			Li et al., 2013
			Risinger et al., 2013a
			Risinger and Mooberry, 2011
			Rohena et al. (2013)
			Peng et al., 2010
1	SCC4 cells	microtubule-stabilizing activity	Risingera et al. (2011)
1, 5	A-10 cells	microtubule-stabilizing activity	Tinley et al. (2003)
68	HCC1937 breast cancer cells	microtubule-stabilizing activity	Du et al. (2019)
1, 5	A549 cells	microtubule-stabilizing activity	Buey et al. (2005)
1	P-388 leukemia cells	cytotoxic activity	Chen et al., 1988a
	HepG2, and Huh7 cells		Tian and He, (2020)
1, 5, 42	MDA-MB-435 and HeLa cancer cell lines	cytotoxic activity	Tinley et al. (2003)
			Risinger et al. (2013a)
18, 27–28, 30–33, 42, 44–46	HeLa cells	cytotoxic activity	Peng et al., 2010
1, 5	Mam17/ADR model	antitumor activity	Risinger et al. (2008)
32, 20, 48–56	MDA-MB-231 breast cancer xenograft model	antitumor activity	Peng et al., 2014
			Risinger et al. (2013b)
66	HeLa, SK-OV-3 cells	cytotoxic activity	Du et al. (2020)
1, 5	SK-OV-3, MDA-MB-435	activity on multidrug-resistant cell lines	Tinley et al. (2003)
1	Pgp overexpressing Mam17/ADR model	activity on multidrug-resistant cell lines	Risinger et al. (2008)
2, 5, 14	wild-type βIII cell line	activity on multidrug-resistant cell lines	Risinger et al. (2008)
42	HeLa βIII-tubulin overexpressing paclitaxel resistant cells	activity on multidrug-resistant cell lines	Balaguer et al. (2019)
1	*Plasmodium berghei*	antimalarial activity	Chen et al. (1988a)
1	*Trypanosoma brucei*	antitrypanosomal activity	Dike et al. (2016)

### Microtubule stabilizing activity

The unique microtubule stabilization mechanism of taccalonolides is imparted by the covalent bonding of the C22–C23 epoxy moiety to tubulin, which causes the density of interphase microtubules to increase, G_2_/M cell accumulation, Bcl-2 phosphorylation, and initiation of apoptosis (Risinger and Mooberry, 2010; Peng et al., 2011; Cao et al., 2018). Thus, as novel microtubule stabilizers, taccalonolides could circumvent clinically relevant forms of drug resistance. Moreover, taccalonolides could enhance the extent of tubulin polymerization without affecting the time required to initiate tubulin polymerization, which made them distinct from other microtubule stabilizers (Peng et al., 2014). In the past few years, many investigations have been performed on the microtubule-stabilizing activity of taccalonolides. Taccalonolides with C23–C26 lactone ring (e.g., taccalonolides A and E) are proved to have moderate or strong microtubule-stabilizing effects on different human cancer cell lines (e.g., HeLa, SCC4, and A-10 embryonic aortic smooth muscle cells).

Risinger *et al.* reported that compound **42** stabilized the plus ends of microtubules more effectively than paclitaxel. The increased resistance of microtubule plus ends to catastrophe may play a role in the observed inability of taccalonolide-induced asters to coalesce during mitosis (Risinger et al., 2014). However, in recent years, more researchers focused on the relative contribution of key tubulin residues and taccalonolide moieties for drug-target interaction. The high-resolution crystallographic data showed the M-loop of **42** was in an unordered conformation. Meanwhile, hydrogen-deuterium exchange experiments indicated that taccalonolides did not promote M-loop stabilization (Risinger et al., 2013a; Wang et al., 2017; Balaguer et al., 2019). Wang *et al.* determined the 2.05 Å crystal structure of the **42**-tubulin complex. The structure revealed that C22–C23 epoxy group of **42** is covalently bound to β-tubulin D226. With a binding of **42**, the M-loop underwent a conformational shift to facilitate tubulin polymerization (Wang et al., 2017). The contact area between taccalonolides and β-tubulin may play a role in microtubule-stabilizing activity. Du *et al.* found the fluorescein moiety of **67** occupied an adjacent binding pocket on β-tubulin close to the M-loop, affording additional interactions with β-tubulin residues via hydrophobic interactions, H bonds, and/or salt bridges. It is suggested the enhanced ability of **61** and **67** to promote microtubule stabilization could be attributed to improving binding affinity to β-tubulin afforded by these additional contacts (Du et al., 2020). These studies firstly reveal the mechanism of the only steroidal natural products, which could bind to the β-subunit of microtubules from the perspective of molecules, atoms, and chemical bonds.

### Anti-tumor activity *in vivo* and cytotoxic activity *in vitro*

Both natural taccalonolides (e.g., **1**, **5**, and **32**) and semi-synthetic taccalonolides (e.g., **42**, **55**, and **66**) have good cytotoxic activity *in vitro* (e.g., HeLa, HepG2, and Huh7 cells) and antitumor activity *in vivo* (e.g., MDA-MB-231 and -435 breast cancer xenograft model). The present review will discuss the progress of cytotoxic and antitumor activities of taccalonolides in recent 6 years.

When HepG2 and Huh7 cells were treated with **1**, the expression of apoptosis-associate protein Bax was up-regulated, whereas Bcl-2 was down-regulated. It was indicated compound **1** could improve the cytotoxicity of sorafenib in hepatocellular cancer by inhibiting the activation of the sonic hedgehog pathway (Tian and He, 2020). Compound **66,** reported by Du *et al.* represented a cell-permeable, fluorogenic probe that combined the potent antiproliferative activities of **42** with excellent fluorescence properties. In HeLa or SK-OV-3 cells, the direct drug-fluorophore conjugation in 66 led to GI50 values of 30–50 nM (Du et al., 2020).

Compounds **1**, **42**, and taccabulin A were combined to evaluate the synergistic antiproliferative effects in MDA-MB-435 and HeLa cancer cell lines. The result showed the CI values were between 0.65 and 0.85. Moreover, the synergy of **42** and taccabulin A was more obvious. The study was the precedent for the combination of microtubule-stabilizing and -destabilizing small molecules in combination chemotherapy (Risinger et al., 2013b). The antitumor action *in vivo* may relate to half-life. Compound **42** exhibited excellent and highly persistent antitumor effects when directly acting on tumors. However, it was no antitumor effects when administered systemically, probably due to the short half-life (8.1 min) *in vivo* (Risinger et al., 2017).

### Activity on drug-sensitive and multidrug-resistant cell lines

The multidrug resistance of cancer cells is evolved by multiple mechanisms, including overexpression of P-glycoprotein (Pgp), multidrug resistance protein 7 (MRP7), and βIII isotype of tubulin (Morris and Fornier, 2008). Unlike paclitaxel, taccalonolides are not substrates of Pgp. Taccalonolides exhibit *in vivo* antitumor efficacy in both drug-sensitive and resistant tumor models. They are still against paclitaxel-resistant tumor cells (Tinley et al., 2003; Risinger et al., 2008). Ola *et al.* found taccalonolides substituted with isovalerate in position C7 or C15, along with epoxy group connected at C22–C23, showed effective and highly persistent antitumor activity in paclitaxel-resistant xenograft model when administered intratumorally, without associated toxicity (Ola et al., 2018). Compounds **1** and **5** completely inhibited cell proliferation and induced cell death in drug-sensitive cell lines SK-OV-3 and MDA-MB-435. These two compounds also induced cytotoxicity in the drug-resistant cell line NCI/ADR (Tinley et al., 2003). Both compound **1** with a total dose of 38 mg/kg, and compound **5** with 86 mg/kg, showed 91% growth inhibition on the Pgp overexpressing Mam17/ADR model, indicating they have excellent antitumor activity *in vivo*. Besides, **5** might be better tolerated than **1** (Risinger et al., 2008).

MRP7 expression induces drug resistance in non-small-cell lung cancer cell lines on paclitaxel treatment. MRP7 expression levels are correlated with both paclitaxel accumulation and sensitivity. Under the treatment of **1**, **2**, **5**, and **14**, HEK-MRP7-C17 and HEK-MRP7-C18 cell lines were more sensitive compared with the control cells (HEK-pcDNA3). Whether MRP7 was overexpressed, compounds **1**, **2**, **5**, and **14** had similar potency in HEK293 cells (Risinger et al., 2008). The result suggested that the ability of taccalonolides to circumvent MRP7-mediated efflux may provide a significant advantage for the treatment of cancer.

Except for Pgp and MRP7 overexpression, βIII-tubulin isotype expression is a main clinical determinant of resistance to tubulin-target therapy. Dose-response curves suggested that wild-type βIII cells (a HeLa-derived cell line that ectopically expresses the human βIII-tubulin gene) were 4.7-fold resistant to paclitaxel as compared with the HeLa cell line. But the βIII-expressing cell line reproducibly showed sensitivity to **2**, **5**, and **14**, suggesting that incorporation of βIII isotype tubulin into mitotic spindles does not confer resistance to taccalonolides (Risinger et al., 2008). Moreover, IC_50_ of **42** in HeLa and HeLa βIII-tubulin overexpressing paclitaxel-resistant cells was 6.2 and 9.6 nM, respectively (Balaguer et al., 2019). These results suggested that covalent bonding to tubulin facilitates overcoming βIII-tubulin-mediated drug resistance.

## Structure and antiproliferative activity relationship

**FIGURE 7:**
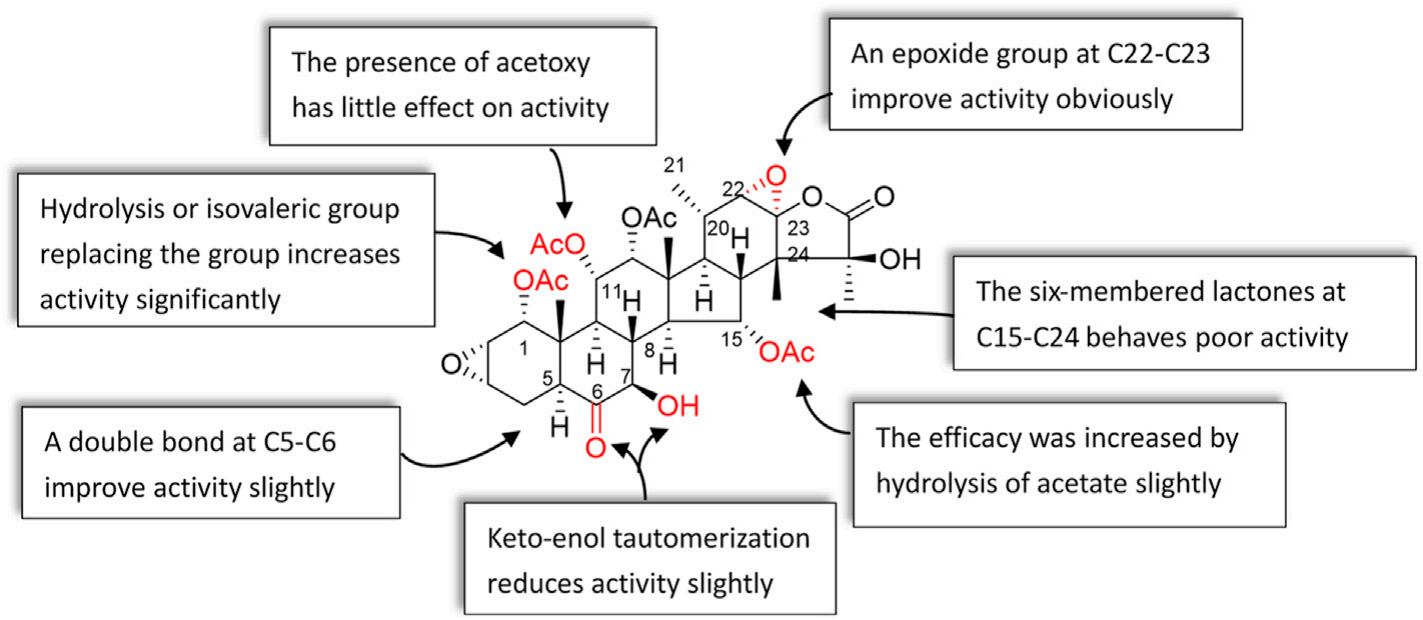
Structure and antiproliferative activity relationship of taccalonolides.

Studying the structure–activity relationship of taccalonolides would be beneficial to designing and synthesizing derivatives as a new generation of antitumor agents with improved physical, chemical, and biological properties. Therefore, we summarize the SAR of taccalonolides, hoping to help identify specific structural moieties crucial for potent biological activities, as well as those that impede optimal cellular effects (Figure 7).

### Effect of C22–C23 structure on activity

The epoxy function in positions C22 and C23 is critical for the potent antitumor effects of the naturally occurring epoxy-taccalonolides. The presence of C22–C23 epoxy group can restore the activity of taccalonolides that are reduced by the C6 ketone (Peng et al., 2014; Danielsson et al., 2017). Compounds containing C22–23 epoxies showed no antiproliferative, cytotoxic, or microtubule bundling activities in cells and did not directly interact with tubulin in biochemical assays (Du et al., 2020). When C22–C23 double bond of **1** and **2** was epoxidized into **32** and **42,** respectively, the antiproliferative activity dramatically increased by 234–743 folds, indicating the epoxidation of C22–C23 double bond significantly enhanced the potency of taccalonolides (Li et al., 2011; Wang et al., 2017; Sanchez-Murcia et al., 2019). After C22–C23 epoxidation, **56** and **57** also showed modest increases of 2–4 folds, and **52** and **53** showed relatively modest 7 and 2 folds increases in potency, respectively (Peng et al., 2014). Surprisingly, **29**, which differed from **1** only by an additional α-hydroperoxyl group at C20, had no antiproliferative or microtubule-stabilizing activities at a concentration up to 50 µM. It was indicated that α-hydroperoxyl group was not optimal for bioactivity and substituent at this site was important (Li et al., 2011). And **43** and **47** have an obvious structural rearrangement in C20–C23 region, but antiproliferative activity or microtubule-stabilizing effects were not observed at concentrations up to 50 µM (Li et al., 2013). In summary, the results highlight the importance of substituents at C20–C23 of taccalonolides.

### Groups at C1 affect the activity

Modifications in position C1 could significantly affect the potency of taccalonolides. Compound **20** with an isovalerate group in position C1 has better antiproliferative potency than compound **18** substituted with an acetoxy group in the same position. The antiproliferative potency of **20** is 38-folds of **18** (Peng et al., 2011). Replacement of the acetoxy group at C1 in **44** with an isovalerate group generates **45**. Similarly, 17-folds increasement in the potency of **45** was observed (Li et al., 2013). Hydrolysis of the acetoxy group at C1 or C15, or retention of the isovalerate group at C1 could significantly improve the biological activity. Modifications at C1 enhanced activity were linked to the data of the crystal structure. Peng *et al.* reported that **55** showed an even higher potency than **42** (Peng et al., 2014). The bulky isovalerate group at C1 of **55** was well-positioned into a hydrophobic pocket surrounded by L217, L230, L275, and F272, which might be the reason for the higher potency than **42** (Wang et al., 2017). These data emphasize the importance of the group in C1 position on the activity of taccalonolides.

### Effect of C5–C8 region on activity

Compound **9** differs from **1** in the positions of C6 and C7: the ketone and hydroxy groups are located in positions C6 and C7 of **1,** respectively, while they are opposite of **9**. The difference resulted in a 9.25-folds decrease in antiproliferative potency of **9**. However, compound **30** contained a C5–C6 enol group and a C7 ketone is more effective than **1**, suggesting that the double bond plays a role in retaining antiproliferative activity. Compared with **1**, **33** possesses a double bond in positions of C7 and C8, leading to the potency increased by 7.4 times, which illustrates that the α, β-unsaturated ketone at C7–C9 might have the best potency. In addition, when a hydroxyl group was added to C7 of **1** to form the rare geminal diol compound **31**, the potency also unchanged (Li et al., 2011). The effect of C5 hydroxyl group on the efficacy of taccalonolides is complicated. Compared with **14**, the C5 hydroxyl group in **44** caused a 4-fold decrease in the effectiveness (Li et al., 2013). However, compared to **1**, the potency of **26** contained a C5 hydroxyl group was increased 44 times (Peng et al., 2011).

### Cellular effects of the C6-fluorescein taccalonolide

The C6 metabolite of **1** decreased the potency, demonstrating the importance of the C6 ketone (Peng et al., 2014). Furthermore, the study showed improvement of antiproliferative activities could be achieved for taxane-based probes by replacement of a β-alanine linker with a shorter glycine linker (Lee et al., 2017). Du *et al.* synthesized the dipivaloyl-protected taccalonolide probe **66** featuring direct conjugation of the fluorescein moiety with the taccalonalide skeleton by an amide bond. Indeed, the antiproliferative potency of **66** was a 50-folds improvement as compared to **63** and <10-folds difference as compared to the untagged **42** against HeLa and SK-OV-3 cell lines (Du et al., 2020). The IC_50_ value of **68** was determined to be 2.5 ± 0.1 µM. This represented a 600-fold decrease in cellular potency as compared to **42**. Additionally, the generation of a functional C6 taccalonolide probe provides a proof of principle for utilizing C6 as a site for conjugation of the taccalonolides for targeted drug delivery (Du et al., 2019).

### Other factors affect the activity of taccalonolides

Taccalonolides modified at C7 or C15 positions were easy to hydrolysis in aqueous solutions, and modification at C25 resulted in the disappearance of biological activity (Ola et al., 2018). Hydrolysis of the C15-acetoxy group in **1** or **5** affords **2** or **14,** respectively. The activity of **2** was 3.1 times higher than that of **1**, while **14** was 6 times higher than that of **5** in HeLa cells, indicating hydrolysis of acetate in C15 position increase the efficacy (Li et al., 2013). The presence of acetoxy in C11 position showed little effect on the efficacy of taccalonolides. Compound **1** differs from **5** in only one substituent: it contains an acetoxy group instead of a hydroxyl group at C11. However, their activities were similar to each other. Similarly, the potency of **2** and **14** show no obvious difference. The taccalonolides contain six-membered lactones connected in positions C15 and C24 that have poor antitumor activities, which is illustrated by the results of **34** and **39.** These two compounds exhibit no antiproliferative activity against various cancer cell lines (Ni et al., 2015).

## Conclusion and perspectives

Drugs that affect microtubule dynamics, including the taxanes and vinca alkaloids, have been a mainstay in the treatment of leukemias and solid tumors for decades (Risinger et al., 2009). As lead compounds with microtubule targeting activities, the structural and biological diversity of taccalonolides provide the directions to develop new antitumor drugs, especially for solid tumors. In addition to paclitaxel-like microtubule-stabilizing activity, taccalonolides exhibit circumvention of paclitaxel resistance, stronger antiproliferation *in vitro*, and antitumor activity *in vivo*. Among them, taccalonolides A and E are more prominent with better microtubule-stabilizing activity and dose-dependent effects on cell cycle distribution and microtubules. Moreover, circumvention of Pgp-mediated drug resistance *in vivo* by them was exciting. These two compounds belong to C23–C26 lactone ring class. Compared with other types of taccalonolides, their better activity suggests more attention should be paid to these C23–C26 lactone ring classes of taccalonolides to find lead compounds with better activity.

Microtubule stabilizers have been confirmed to play important roles in clinical cancer therapy. They would be more and more widely used in clinics for cancer treatments. As new microtubule stabilizers with multiple structures, taccalonolides possess unique mechanisms of stabilizing microtubules like paclitaxel, along with weaker toxicity than colchicine, as well as better drug resistance than paclitaxel. With SAR of taccalonolides being revealed, new lead compounds from taccalonolides used to replace paclitaxel-resistant microtubule stabilizers would be gradually developed. Moreover, it will refocus attention on the source of anticancer drugs to natural products to promote the development of drugs based on natural active products if taccalonolides-like compounds would have been developed into anticancer drugs.
